# A Clinical Prediction Formula for Apnea-Hypopnea Index

**DOI:** 10.1155/2014/438376

**Published:** 2014-10-01

**Authors:** Mustafa Sahin, Cem Bilgen, M. Sezai Tasbakan, Rasit Midilli, Ozen K. Basoglu

**Affiliations:** ^1^Department of Otorhinolaryngology, Diskapi Yildirim Beyazit Research and Training Hospital, Irfan Bastug Street, Dıskapi, 06110 Ankara, Turkey; ^2^Department of Otorhinolaryngology, Ege University School of Medicine, İzmir, Turkey; ^3^Department of Chest Diseases, Ege University School of Medicine, İzmir, Turkey

## Abstract

*Objectives*. There are many studies regarding unnecessary polysomnography (PSG) when obstructive sleep apnea syndrome (OSAS) is suspected. In order to reduce unnecessary PSG, this study aims to predict the apnea-hypopnea index (AHI) via simple clinical data for patients who complain of OSAS symptoms. *Method*. Demographic, anthropometric, physical examination and laboratory data of a total of 390 patients (290 men, average age 50 ± 11) who were subject to diagnostic PSG were obtained and evaluated retrospectively. The relationship between these data and the PSG results was analyzed. A multivariate linear regression analysis was performed step by step to identify independent AHI predictors. *Results*. Useful parameters were found in this analysis in terms of body mass index (BMI), waist circumference (WC), neck circumference (NC), oxygen saturation measured by pulse oximetry (SpO_2_), and tonsil size (TS) to predict the AHI. The formula derived from these parameters was the predicted AHI = (0.797 × BMI) + (2.286 × NC) − (1.272 × SpO_2_) + (5.114 × TS) + (0.314 × WC). *Conclusion*. This study showed a strong correlation between AHI score and indicators of obesity. This formula, in terms of predicting the AHI for patients who complain about snoring, witnessed apneas, and excessive daytime sleepiness, may be used to predict OSAS prior to PSG and prevent unnecessary PSG.

## 1. Introduction

OSAS is one of the most significant health problems in middle-aged people and leads to daytime sleepiness and cognitive deficiencies as well as many systemic diseases. It is a highly prevalent disorder, and at least 4% of middle-aged males and 2% of middle-aged females are estimated to be affected [1]. Diagnosis of OSAS is essentially performed via patient history, clinical examination, and some anthropometric measurements. Polysomnography (PSG) helps to establish a definite diagnosis. Overnight in-laboratory PSG is used most widely to confirm or to refute a suspected OSAS. However, in-lab PSG's limited availability, its high cost, and time- and labor-consuming nature are its disadvantages. The waiting lists of sleep clinics for PSG are quite long, and it is difficult to perform PSG on all patients suspected of OSAS [2]. Thus, many studies that aim to diagnose OSAS via clinical findings and easily applicable tests have been carried out. To this aim, multivariate clinical prediction formulas have been developed using mathematical modeling to assess ambulatory PSG, nap or half night PSG, morphometric analysis, self-reported symptoms, and questionnaires. While declared prediction models using logistic regression have high sensitivity (more than 85%), their specificity is low (less than 55%) [3]. Because a low cost and less time-consuming diagnostic method is needed, this study aims to evaluate the predictive values of symptoms and anthropometric, laboratory, and physical examination findings in order to determine a formula to detect OSAS in patients earlier and to reduce the constantly increasing PSG density in our sleep centers.

## 2. Material and Methods

### 2.1. Methods

#### 2.1.1. Study Population

In this observational study, we retrospectively evaluated 390 consecutive, unselected subjects who were referred to the sleep laboratory of a university hospital to evaluate presumed sleep-disordered breathing and who had undergone PSG. Each individual included in this study was referred to sleep laboratory by otolaryngology department after detailed ear, nose, and throat examination which was performed by the same specialist. Demographic data (age, gender, smoking history, and alcohol and psychotropic drug use), anthropometric measurements (height, weight, body mass index, and circumferences of neck, waist, and hip), and medical history were evaluated.

BMI, the most commonly used method to measure obesity, was calculated by dividing weight in kilograms by the square of height in meters (kg/m^2^). Neck circumference was measured in centimeters at the level of the cricothyroid membrane. Waist circumference was measured in centimeters at the level above the iliac crest. Hip circumference was measured in centimeters while patients were standing still and their feet were fairly close and at the level of the point where the maximum circumference over the buttocks was measured.

After asking patients about their complaints, the following parameters emerged: snoring, witnessed apnea, the existence of nasal obstruction, smoking, and alcohol use. Subjective daytime sleepiness was assessed by using the Turkish version of the Epworth Sleepiness Scale (ESS). Scores higher than 10 were considered to be sleepiness. Pulmonary function tests (including spirometry and flow volume curves), chest X-rays, pulse oximeters in polyclinics to evaluate peripheral oxygen saturation (plus MED-pulse oximeter, plus 50-DL, Contec Medical Systems), arterial blood gas analysis, and a full-night in-laboratory PSG were performed on all subjects.

#### 2.1.2. Ear Nose Throat Examination

Detailed otolaryngologic physical examinations were performed by the same specialist. Parameters obtained in these examinations were included in statistical evaluations and included the following: protrusion and retrusion of the mandibles, tonsil sizes, the relationship between the soft palate and the neutral position of the tongue, the tongue-base size, nasal septum deviation, the inferior turbinate size, the endoscopic nasal cavity, and nasopharynx examinations using a 0° rigid endoscope, endoscopic larynx, and hypopharynx examinations using a 70° rigid endoscope (4 mm, 18 cm, Korl Storz Hopkins, Tuttlingen, Germany).

Mandibular retrognathism was evaluated in accordance with the position of the pogonion when the virtual imaginary line between the vermilion line and the chin on a patient sitting in a Frankfort horizontal position is taken into consideration. The examinations of the oral cavity started with an inspection of the relative position of the hard and of the soft palate to the tongue inside the mouth, without protrusion using the modified Mallampati index (MMI), ranging from Class I to Class IV, with Class I representing the highest visibility (the tonsils, the pillars, and the soft palate being visible) and Class IV representing the lowest level of visibility (only the hard palate being visible) of the posterior oropharynx [4]. The tonsils were classified by degree in accordance with hypertrophy, from Degree I to Degree IV as follows: tonsils in the tonsillary fossa and they are barely seen behind the anterior pillars were Grade I, tonsils that occupied 25% of the oropharynx were Grade II, tonsils that occupied 50% of the oropharynx were Grade III, and tonsils that occupied at least 75% of the oropharynx were Grade IV, if they meet in the midline. Tongue-base sizes were ranked between Grades 1 and 3. The tongue was classified as Grade 1 when the vallecula was partially visible in an examination using a 70° rigid endoscope, while the tongue was in an easy position within the mouth; it was classified as Grade 2 when the vallecula was invisible and the tongue base touched the epiglottis; and when the tongue base pushed the epiglottis, it was classified as Grade 3. Nasal examinations were performed via anterior rhinoscopy and a 0° rigid endoscope, and internal nasal pathways were evaluated following research on pathology, such as septum deviation, turbinate hypertrophy, and intranasal obstructive lesions, such as polypus. The movement of obstructive lesions and the vocal cords in the larynx and in the hypopharynx was evaluated following a larynx examination via a 70° rigid endoscope.

#### 2.1.3. Polysomnography

All subjects underwent a full overnight in-laboratory diagnostic PSG (Compumedics E Series, Australia or Alice 5 Diagnostic Sleep System, Philips, Respironics, USA). Electroencephalography electrodes were positioned according to the international 10–20 system. PSG consisted of monitoring of sleep by electroencephalography, electrooculography, electromyography, airflow, and respiratory muscle effort and included measures of electrocardiographic rhythm and blood oxygen saturation. Thoracoabdominal plethysmograph, oronasal temperature thermistor, and nasal-cannula pressure transducer system were used to identify apneas and hypopneas. Transcutaneous finger pulse oximeter was used to measure oxygen saturation. Sleep was recorded and scored according to the standard method [5]. AHI was the sum of the number of apneas and hypopneas per hour of sleep. OSAS was defined as an AHI of 5 events/h and the presence of clinical symptoms, for example, excessive daytime sleepiness, loud snoring, witnessed apneas, and nocturnal choking, or AHI of 15 events/h without any OSAS symptoms [6]. Besides, an AHI of <5 events/h was considered within normal limits. No split-night studies were performed.

#### 2.1.4. Statistical Analysis

SPSS version 17.0 was used for statistical analysis. First, the correlation between the AHI and all of the variables included in the study was analyzed in order to identify variables to be used to predict the AHI. Mann-Whitney *U* test was performed to evaluate the statistical difference between AHI values of men and women. Following this test it was not a statistically significant difference between men and women according to AHI values (*P* = 0.190). Therefore different models were not developed regarding the gender. Prior to the correlation analysis, we identified whether these variables provided parametric assumptions. We found in the results of the Kolmogorov-Smirnov test that variables did not demonstrate normal distribution. Spearman's rank order correlation analysis was performed because variables did not provide normal distribution as required. Following this analysis, variables that were identified to have had a correlation with the AHI were integrated into the regression analysis. Each variable was integrated into the regression in order to identify how these variables as a whole affected the AHI via a step-by-step, multiple linear regression analysis. When the *P* value was <0.05, it was regarded as statistically significant.

## 3. Results

The demographic data (age, gender, smoking history, and alcohol and psychotropic drug use), anthropometric measurements (height, weight, body mass index, and circumferences of the neck, waist, and hips), and medical history variables are shown in Table 1.

Variables that were integrated into the regression analysis after we found that they were correlated with the AHI in Spearman's rho test were the BMI, NC, WC, hip circumference (HC), smoking rate in packages/year, force vital capacity (FVC), forced expiratory volume ratio in one second (FEV1%), FEV1/FVC ratio, Pao2, PaCO2, ESS score, oxygen saturation level via a pulse oximeter, the Epworth Scale score, and tonsil size. Correlation coefficients of these variables in nonparametric Spearman's rank order correlation analysis are shown in Table 2.

Thereafter, each variable was integrated into the regression analysis in order to identify how these variables as a whole affected the AHI via a step-by-step multiple linear regression analysis. In the seventh phase, the explanatory power of the model reached its peak: *R* ^2^ = 0.682. Therefore, the following model was developed to predict the AHI: AHI prediction = (0.797 × BMI) + (2.286 × NC) − (1.272 × SpO_2_) + (5.114 × TS) + (0.314 × WC). The most significant variables in the regression model developed to the AHI prediction via a step-by-step multilinear regression analysis are shown in Table 3.

**Figure 1:**
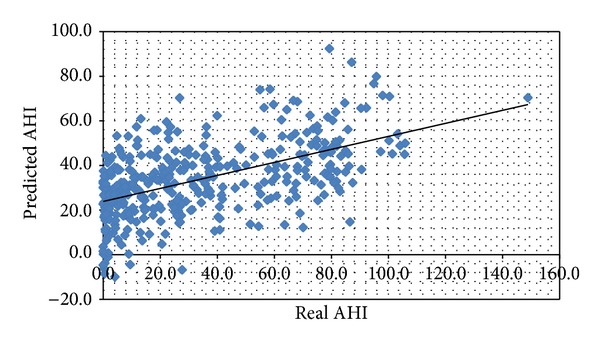
Distribution of predicted AHI values regarding real AHI values.

We found this model to be statistically significant (*F* = 155.348, *P* < 0.05). According to this formula, 68.2% of the variation in the AHI could be explained via these variables, while 32.8% could be attributed to other variables. Bland Altman plot comparing real AHI values obtained from PSG and the predicted AHI values obtained from the prediction formula is shown in Figure 1.

## 4. Discussion

Some studies have shown that data based on medical history and the findings of the physical examination may be useful to detect OSAS in patients. Hoffstein and Szalai obtained a sensitivity of 60% and a specificity of 63% in the detection of OSAS in patients in a study that included 594 patients and aimed to analyze the statistical relevance of data related to medical history and physical examinations [7]. In our study to predict the AHI, a clinical formula with 0.682 explanatory power was found including these parameters: body mass index, neck circumference, waist circumference, peripheral oxygen saturation, and tonsil size.

Sleep apnea, despite being the most common reason for EDS, was not considered to be a useful clinical feature for detecting OSAS in patients because 30 to 50% of the society claim moderate to sleepiness [1, 2]. In addition, other sleep disorders and diseases may cause EDS. Bausmer et al. did not find a significant correlation between the ESS score and the AHI [8]. In a study analyzing the relation between clinical parameters and OSAS on a group of 80 people, similar to our present study, no significant correlation was found between subjective sleepiness measured via the ESS and the AHI [9].

Obesity is an important risk factor for OSAS, and 70% of OSAS patients suffer from it. The most commonly used measurements in the diagnosis of obesity are BMI, WC, and HC. Bouloukaki et al. stated that BMI was a better indicator than other obesity indicators, such as waist-to-hip ratio, in OSAS predictability [10]. In many studies, NC, which is related to fat deposition around the upper airway, was defined as an important predictor factor for OSAS [3]. The value of NC in predicting OSAS is still controversial. In a study of 2,690 patients, Bouloukaki et al. reported that NC was the most significant correlate with the AHI [10]. In this study, similar to the findings in previous studies, NC and BMI were found to be significant parameters for the AHI prediction.

It is a challenging issue for otolaryngologists to identify and to improve the location of obstruction level(s) in the upper airway (UAW) in patients suspected of OSAS. The UAW is assumed to be narrower and/or more prone to collapse multilevelly in OSAS patients. The relationship between anatomic abnormalities and the severity of OSAS has not been well established in previous studies because UAW abnormalities are not the only causative factors [11]. In this study just TS was found significant in predicting the AHI among the physical examination findings of UAW, we only found TS to be significant in predicting the AHI. It is generally acknowledged that tonsillar hypertrophy causes airway obstruction. TS has been related to the AHI in a few different studies. Friedman revealed a relation between the increase in TS and the AHI through grading tonsil sizes in OSAS patients [4]. Enlarged tonsils are associated with OSAS, and surgical removal is thought to be an important part of the treatment. Cahali et al. reported that the tonsil grade has a strong correlation with the AHI in OSAS patients [12].

There is no consensus regarding the effect of nasal airway blockage on OSAS pathogenesis and the severity of the disease. While some authors argued that the nasal passage affects OSAS development and is related to AHI, other authors claimed the converse [13]. In this study, no statistically significant correlation between nasal obstruction and the AHI was detected.

The Mallampati index, which was defined by anesthesiologists in 1985 to identify difficult intubation, was later suggested as an MMI to be used in predicting OSAS [4]. This index is used to evaluate oropharyngeal structures and relative tongue size. Some studies reported that relative tongue size may be related to AHI. Liistro et al. [14] and Schellenberg et al. [15] concluded that MMI and tongue-base hypertrophy were related to AHI. However, in this study, similar to Dreher et al.'s findings, we found no significant relationship between tongue-base hypertrophy, MMI, and the AHI [16].

In many studies, pulmonary function tests were not included in the data to detect OSAS predictors. Herer et al. evaluated 102 obese patients by clinical features, pulmonary function tests, arterial blood gas tensions, and oximetry for prediction of OSAS prior to PSG and stated that none of these data is sufficient to prove the existence of OSAS [17]. In a study which attempted to develop a predictive index for OSAS based on pulmonary function parameters, conducted with obese snorers suspected of OSAS, it is found out that daytime oxygen saturation contributed to the model significantly. It was argued that this model could help decrease redundant PSG applications by 38% [18]. In our study, among spirometric parameters, the FVC, FEV1%, and FEV1/FVC ratios were found to be correlated with the AHI, but these parameters were eliminated in the regression analysis and did not come out in the AHI predictive model as independent variables. The relationship between the levels of AHI and desaturation were also argued, and no strong significance was identified about this issue. Since it is easier to apply and it is more cost-effective, transcutaneous pulse oximetry has become more and more widely used in initial screenings of OSAS. Mulgrew et al. stated that PSG applied to patients suspected of OSAS is by no means superior to ambulatory methods based on oximeter readings combined with clinical data [19]. In their study of 275 patients, Chiner et al. found that a preliminary pulse oximeter may decrease redundant PGS ratios by 38% in a group of patients where the AHI is more than 15 [20]. On the other hand, no desaturation can be seen among hypopnea period and in cases where upper airway resistance has increased even when oxygen desaturation is prevalent in obstructive apnea. Therefore, an oximeter may be useful on its own, particularly as far as higher average levels of OSAS are concerned and where they cannot rule out the diagnosis [21]. Because putting all patients through overnight oximeters conflicts with the practicability of our study, we analyzed the pulse oximeters and oxygen saturations in peripheral blood in policlinic environment and found that evaluation was one of the most significant parameters in the AHI prediction model.

However, such formulas include different variables within different populations, whose differences may be a significant limitation of this study. Therefore, this method may not be sufficient since a screening technique and its validity need to be further tested in other series and would need to include more patients. It is our next aim to design a prospective study to evaluate the predictability of the formula obtained from this study.

As a result, a mathematical model created via data obtained through simple office procedures to predict OSAS may reduce redundant PSG and result in more rapid diagnoses and treatment processes. The potential advantage of this approach is its ease of use, its cost effectiveness, its applicable nature in office environments, and its contribution to more rapid decisions. Validity of this prediction formula needs to be further tested in other prospective researches. It is not possible to make a clear clinical statement about this formula yet. Validated prediction models, which will be created via collective studies by more sleep centers and with more groups of patients, are needed because no single, sufficient model has yet been described.

## Figures and Tables

**Table 1:** Characteristics of the study population.

Variables	Study population (*n* = 390)
Age (year)∗	50.1 ± 11.1
Sex (*n*, %)	Male: 289 (73.9%)
	Female: 101 (26.1%)
Current Smoker (*n*, %)	116 (29.7)
Alcohol Consumption (*n*, %)	67 (17.2%)
Comorbidities (*n*, %)	
Hypertension	156 (40 %)
Diabetes mellitus	76 (19.5 %)
Coronary artery disease	31 (7.9 %)
BMI (kg/m^2^)∗	30.8 ± 5.5
Neck Circumference (cm)∗	40.9 ± 4.2
Waist Circumference (cm)∗	106.1 ± 14.6
Hip Circumference (cm)∗	110.1 ± 11.2
Epworth Sleepiness Score∗	9.8 ± 6.0
Apnea-hypopnea index (/hour)∗	33.2 ± 30.3

BMI: Body mass index, NC: Neck circumference, WC: Waist circumference, HC: Hip circumference, SpO_2_: Oxygen saturation, FVC: Forced vital capacity, FEV1%: Forced expiratory volume ratio in one second, ESS: Epworth Sleepiness Scale's score, TS: Tonsil size. ∗Values are expressed as mean (SD).

**Table 2:** Variables identified to be related to AHI in Spearman's rho test and their correlation coefficients.

Variable	Correlation coefficient	Variable	Correlation coefficient
BMI	0.491	FVC	−0.149
NC	0.358	FEV1%	−0.101
WC	0.371	FEV1/FVC	−0.085
HC	0.112	PaO_2_	−0.203
Smoking	0.190	PaCO_2_	0.161
ESS	0.103	SpO_2_	−0.242
		TS	0.431

**Table 3:** Parameters used to identify independent predictors of apnea-hypopnea index.

Parameter	Beta	*T*	*P* value
Body mass index	0.797	2.132	0.034
Neck circumference	2.286	6.696	<0.0001
Oxygen saturation	−1.272	−9.094	<0.0001
Waist circumference	0.314	2.274	0.24
Tonsil size	5.114	2.261	0.024
